# Nutrigenomics of Vitamin D

**DOI:** 10.3390/nu11030676

**Published:** 2019-03-21

**Authors:** Carsten Carlberg

**Affiliations:** School of Medicine, Institute of Biomedicine, University of Eastern Finland, FI-70211 Kuopio, Finland; carsten.carlberg@uef.fi

**Keywords:** Vitamin D, VDR, nutritional epigenomics, transcriptome, immune system, evolution

## Abstract

Nutrigenomics studies how environmental factors, such as food intake and lifestyle, influence the expression of the genome. Vitamin D_3_ represents a master example of nutrigenomics, since via its metabolite 1α,25-dihydroxyvitamin D_3_, which binds with high-affinity to the vitamin D receptor, the secosteroid directly affects the epigenome and transcriptome at thousands of loci within the human genome. Vitamin D is important for both cellular metabolism and immunity, as it controls calcium homeostasis and modulates the response of the innate and adaptive immune system. At sufficient UV-B exposure, humans can synthesize vitamin D_3_ endogenously in their skin, but today’s lifestyle often makes the molecule a true vitamin and micronutrient that needs to be taken up by diet or supplementation with pills. The individual’s molecular response to vitamin D requires personalized supplementation with vitamin D_3_, in order to obtain optimized clinical benefits in the prevention of osteoporosis, sarcopenia, autoimmune diseases, and possibly different types of cancer. The importance of endogenous synthesis of vitamin D_3_ created an evolutionary pressure for reduced skin pigmentation, when, during the past 50,000 years, modern humans migrated from Africa towards Asia and Europe. This review will discuss different aspects of how vitamin D interacts with the human genome, focusing on nutritional epigenomics in context of immune responses. This should lead to a better understanding of the clinical benefits of vitamin D.

## 1. Introduction

Nutrigenomics is a research field that developed some 15 years ago after the completion of the human genome [1,2]. Human diet is a complex mixture of biologically active molecules, some of which (i) have a direct effect on gene expression, (ii) modulate, after being metabolized, the activity of a transcription factor, or (iii) stimulate a signal transduction cascade that ends with the induction of a transcription factor [3]. Nutrigenomics aims to describe, characterize, and integrate the interactions between dietary compounds and genome-wide gene expression. Genomic loci related to metabolic and/or regulatory pathways may provide a molecular explanation of how a dietary compound mediates its effects and whether it influences the risk of diet-related diseases.

The discipline of epigenomics studies the effect of environmental events on the expression of the genome [4]. Diet is the main environmental exposure to the human body and represents the key daily lifestyle decision that every individual makes. Thus, a central goal of epigenomics is to get insight how dietary molecules affect the epigenome and by this gene expression [5]. Thus, nutrigenomics and epigenomics have a joint focus. Information provided by both fields may allow developing strategies to permit appropriate lifestyle decisions will lead to healthy, disease-free aging [6].

Next-generation sequencing technologies, such as RNA sequencing, chromatin immunoprecipitation followed by sequencing (ChIP-seq), and formaldehyde-assisted identification of regulatory elements followed by sequencing (FAIRE-seq), allow the unbiased assessment of genome-wide mRNA expression, transcription factor binding, histone modifications, and chromatin accessibility [7]. Large research consortia, such as ENCODE (www.encodeproject.org) and Roadmap Epigenomics (www.roadmapepigenomics.org), collected huge amounts of data on the basal state of more than hundred human cell lines [8] as well as on about the same number of primary human tissues and cell types [9], respectively.

Recent longitudinal molecular assessments of individuals in relation to their lifestyle decisions, such as the Personalized Omics Profiling Project [10,11] and the Pioneer 100 Wellness Project [12], had a clear nutrigenomics component. When individuals are classified according to the features of their lifestyle, metabolic pathways, and (epi)genetic variation, tailored diets, referred to as personalized nutrition [13], can be suggested for early therapeutic intervention. Interestingly, the use of dietary compounds for a specific therapy dissolves the distinction between food and drugs.

This review will discuss nutrigenomic aspects of vitamin D, which are based on epigenome- and transcriptome-wide actions of the micronutrient that were observed in vitro in human cell lines as well as in vivo in the context of human vitamin D intervention studies.

## 2. Vitamin D_3_

**Figure 1:**
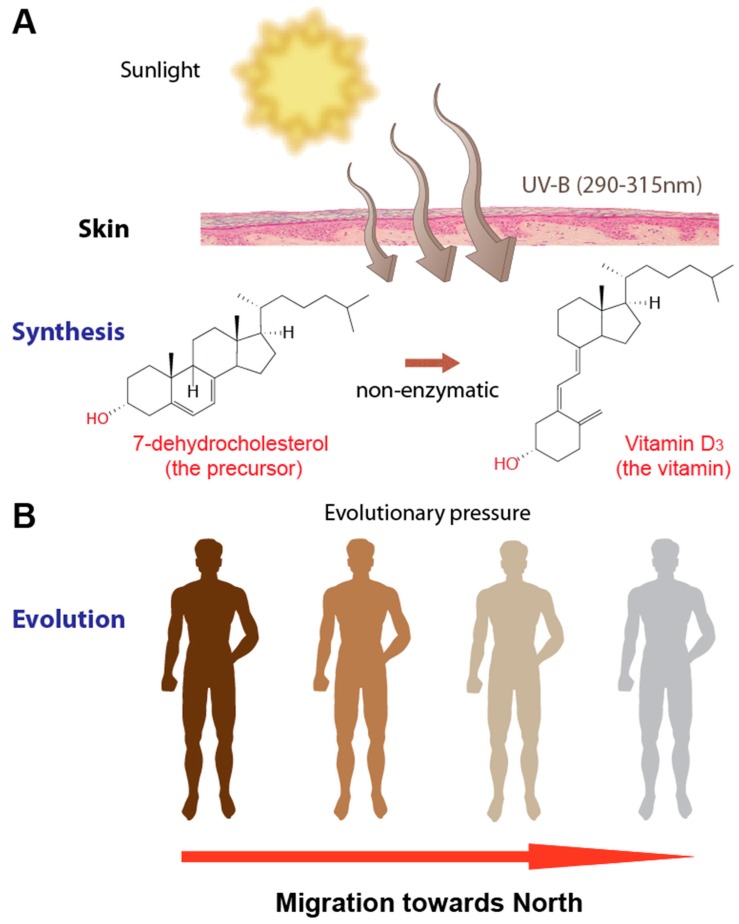
Vitamin D and human skin. Vitamin D_3_ is synthesized endogenously in UV-B exposed human skin (**A**). During the past 10–30,000 years this essential need to synthesize vitamin D_3_ was an evolutionary driver for skin lightening of modern humans migrating from Africa towards Europe and Asia (**B**).

In the past, the primary medical focus on diet was to avoid nutrient deficiency diseases, such as scurvy and rickets, where patients lack the vitamins C and D, respectively. This is illustrated by the use of sunlight and ultraviolet (UV) radiation exposure as an efficient therapy of rickets, which is a childhood disease of bone malformation, as well as of tuberculosis, which is an infectious disease caused by the intracellular bacterium *Mycobacterium tuberculosis*. In parallel, some 100 years ago, the rickets curing ingredient of cod-liver oil was identified and termed “vitamin D”, because it was the vitamin to be named fourth in line [14]. These observations were combined, when many years later it was found that humans can synthesize vitamin D_3_ in their UV-B (290-315 nm) exposed skin [15]. The energy of the UV radiation is used in a non-enzymatic reaction to open the B-ring of the cholesterol precursor 7-dehydrocholesterol, creating the thermodynamically unstable molecule pre-vitamin D_3_, which then further isomerizes into vitamin D_3_ (Figure 1A).

The ability to endogenously synthesize vitamin D_3_ implies that the term “vitamin” may not be correctly used. However, since nowadays humans tend to stay preferentially indoors and cover their skin by textile outdoors [16], insufficient UV-B exposure results in low endogenous vitamin D_3_ production, so that for a huge proportion of the human population, the molecule is an essential micronutrient. Since the average human diet does not contain much vitamin D, worldwide, an estimated number of more than a billion individuals are vitamin D deficient [17]. Fatty fish, such as tuna, sardines, salmon, and mackerel, are the best dietary sources of vitamin D followed by liver, cheese, egg yolks, and mushrooms. Moreover, in some countries, foods, such as milk and other dairy products, orange juice, cereals, and margarines, are fortified with vitamin D. In addition, direct vitamin D supplementation via pills or oily drops is recommended in many countries [18].

## 3. Vitamin D and Human Genetic Adaptation

Some 100,000 years ago, anatomically modern humans (*Homo sapiens sapiens*) lived only in Africa and their skin was dark, because it better protects against sunburns and skin cancer. Dark skin also prevents the photodegradation of circulating folate, which is an important methyl-group donor being important, e.g., for DNA methylation during embryogenesis. The latter aspect may have been the main reason why, approximately one million years ago, human skin became highly pigmented, when the ancestors of modern humans lost most of their body hair, in order to better sweat during endurance physical activity, such as hunting [19]. Interestingly, when modern humans arrived in Europe some 40,000 years ago, the reverse process happened, i.e., their skin turned pale again (Figure 1B).

The main evolutionary driver of this decreased skin pigmentation was the need for sufficient endogenous vitamin D_3_ production in less sunny Europe. The lightest skin is found in the Nordic countries, representing a geographic region where humans live most distant from the equator. Thus, vitamin D influences the human genome not only via VDR-mediated gene regulation, but did act as an evolutionary driver of genome adaptation in terms of skin lightening. Having dark skin when living permanently at northern latitudes would have caused vitamin D_3_ deficiency, resulting in reduced bone strength and a defective immune system. For example, nowadays, the tuberculosis rate of individuals with dark skin living distant from the equator is significantly higher than that of people with light skin [20].

More than 30 genes are known to influence the pigmentation of melanocytes, but single nucleotide polymorphisms (SNPs) affecting the genes *KITLG* (encoding for a ligand of the receptor tyrosine kinase KIT), *TYRP1* (a tyrosine converting enzyme), *SLC24A5*, and *SLC45A2* (both are ion transporters) had the main impact on skin lightening during the past 10–30,000 years [21,22]. Interestingly, Neanderthals and modern Europeans and Asians evolved light skin independently from each other. Taken together, skin lightening due to the need for vitamin D_3_ synthesis in geographic regions with lower levels of UV-B radiation is a key example in nutrigenomics demonstrating how the need for a gene-regulating micronutrient has directed human evolution.

## 4. Vitamin D and Its Receptor in Metabolism and Immunity

**Figure 2:**
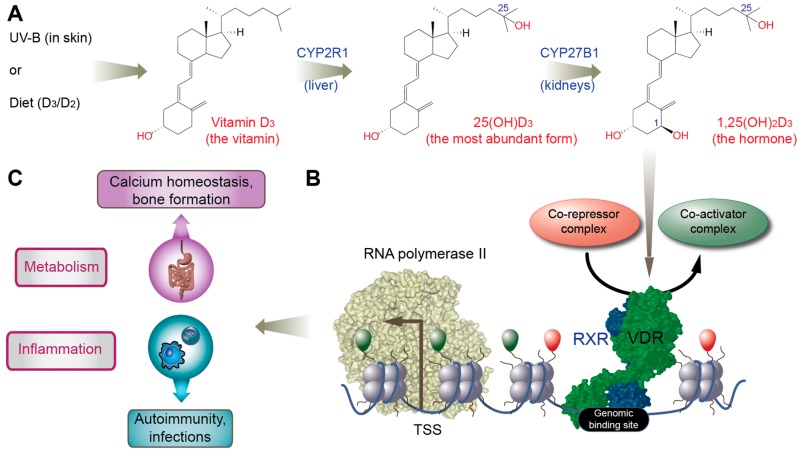
Gene regulation by vitamin D and its physiological impact. Vitamin D_3_, produced endogenously in skin or taken up by diet or supplementation, is converted in the liver into 25(OH)D_3_ and then in the kidneys into the high-affinity vitamin D receptor (VDR) ligand 1,25(OH)_2_D_3_ (**A**). Ligand-activated VDR binds genome-wide to some 5–20,000 sites within accessible chromatin (**B**). Some of these epigenome-wide effects translate into the changes of the transcriptome, i.e., the activation (or repression) of vitamin D target genes. The main physiological processes regulated by these genes are cellular metabolism, such as calcium homeostasis important for bone formation, and the regulation of innate and adaptive immunity, such as improving the response to infectious microbes, reducing chronic inflammation and preventing autoimmune disease (**C**).

In the liver, the biologically inert molecule vitamin D_3_ is converted to 25-hydroxyvitamin D_3_ (25(OH)D_3_, Figure 2A), which is the most stable and abundant vitamin D metabolite in human serum, and is traditionally used as a biomarker for the vitamin D status of individuals [23]. Further hydroxylation at carbon 1 creates 1α,25-dihydroxyvitamin D_3_ (1,25(OH)_2_D_3_, Figure 2A), which acts as an endocrine hormone by serving as a high-affinity ligand (k_D_ 0.1 nM) to the transcription factor vitamin D receptor (VDR, Figure 2B) [24]. The main source of the endocrine production of 1,25(OH)_2_D_3_ are proximal tubule cells of the kidneys, but in a paracrine or autocrine fashion, monocytes, macrophages and dendritic cells of the innate immune system, osteoblasts within bones, and keratinocytes of the skin are also able to produce the hormone [25].

The well-known physiological role of vitamin D is the support of bone mineralization via controlling calcium homeostasis (Figure 2C) [26]. This function fits well with the metabolism-focused profile of some members of the nuclear receptor superfamily, to which VDR also belongs [27]. Comparable members of the superfamily are retinoic acid receptor (RAR) α/β/γ activated by the vitamin A derivative retinoic acid, peroxisome proliferator-activated receptor (PPAR) α/δ/γ binding fatty acids and liver X receptor (LXR) α/β recognizing oxysterols [28]. VDR, RAR, PPAR and LXR act as sensors of the respective micro- and macronutrients, in order to adapt gene expression profiles in metabolic organs as well as in cells of the immune system (Figure 2C). Since most immune cells have a rapid turnover, they are able to show a maximal adaptive response to environmental changes. Macrophages and dendritic cells, as well as their precursors, monocytes, coordinate metabolic, inflammatory, and general stress-response pathways via changes of their transcriptome profile and respective subtype specification [29]. Thus, lipid sensing and signaling via key nuclear receptors has an important role in the differentiation and epigenetic programming of immune cells, indicating that metabolism and immunity are more closely linked than initially assumed.

Via VDR, vitamin D modulates the activity of both the innate and adaptive immune system [30]. For example, in monocytes, vitamin D stimulates the efficient recognition of bacterial pathogens via toll-like receptors (TLRs) [31] and in macrophages, it inhibits the proliferation of *M. tuberculosis* [32]. Notably, tuberculosis is the most disastrous infectious disease that has ever occurred in humans, with an estimated death toll of more than one billion over the past 60,000 years [33]. TLR-triggered expression of vitamin D target genes encoding for the antimicrobial peptides cathelicidin and defensin beta 4A enables killing intracellular *M. tuberculosis* [34]. For this reason, sunlight or artificial UV-B exposure are efficient treatments of tuberculosis. In contrast, vitamin D deficiency is associated with an increased risk of developing clinical tuberculosis. Moreover, vitamin D has been shown to also prevent other type of microbial infections, such as those of the respiratory or urinary tract [35,36].

VDR is a key transcription factor in the differentiation process of myeloid progenitors into monocytes and granulocytes [37]. Moreover, VDR and its ligands antagonize pro-inflammatory transcription factors, such as NF-AT, AP-1, and NF-κB, in T cells, which results in the decreased expression of cytokines, such as IL2 and IL12 [38]. In addition, vitamin D inhibits differentiation, maturation, and the immunostimulatory capacity of dendritic cells via the repression of genes encoding for the different variants of the major histocompatibility complex and its co-stimulatory molecules CD40, CD80, and CD86 [39]. The resulting immune tolerance-inducing phenotype of dendritic cells leads to the induction of regulatory T cells down-regulating the activity other cells of the immune system. This is the central mechanism of how vitamin D dampens chronic inflammation and autoimmunity in diseases such as inflammatory bowel disease [40] and multiple sclerosis [41].

Interestingly, the imprinting of dendritic cells with tolerogenic properties involves the reprogramming of their glucose metabolism via up-regulation of the vitamin D target gene 6-phosphofructo-2-kinase/fructose-2,6-biphosphatase 4 (*PFKFB4*), which encodes for a key glycolytic enzyme [42]. Another glycolytic enzyme, fructose-bisphosphatase 1 (FBP1), is encoded by one of the most responsive primary vitamin D target genes of the monocytic cell line THP-1 [43]. This suggests that, also in vitamin D signaling, immunomodulatory and metabolic functions are more closely linked than initially assumed and probably evolved in parallel. Accordingly, the concept of immunometabolism suggests that metabolism is the key process determining the phenotype and function within immune cell subsets [44].

## 5. Nutritional Epigenomics

**Figure 3:**
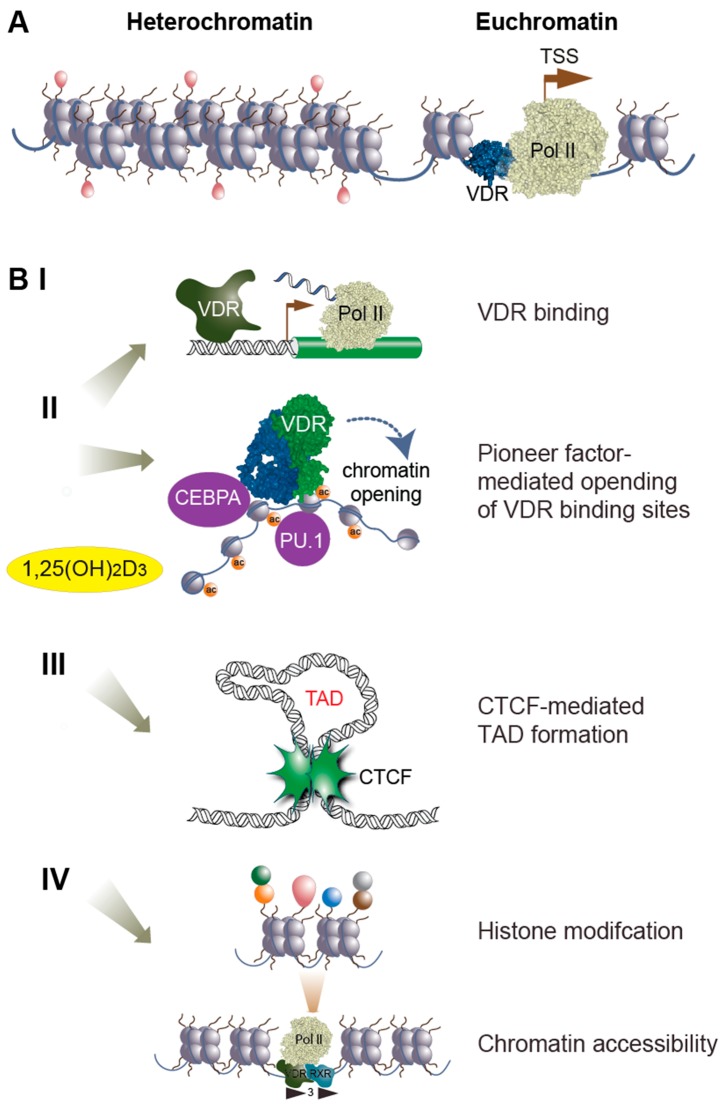
Vitamin D and the epigenome. Chromatin is segregated into non-accessible heterochromatin and euchromatin, where VDR can find its genomic binding sites (**A**). Vitamin D can influence the epigenome in multiple ways (**B**), such as increasing genomic VDR binding (I), affecting the binding of pioneer transcription factor (II), influencing CCCTC binding factor (CTCF) binding and the formation of topologically associated domains (TADs) (III) and changing histone modifications and chromatin accessibility (IV).

The three-dimensional complex of genomic DNA and nucleosome-forming histone proteins is called chromatin [45]. It is categorized into less densely packed euchromatin, which easily accessible to transcription factors and other nuclear proteins, and compact heterochromatin, which is a functionally repressed state [46] (Figure 3A). Post-translational histone modifications and DNA methylations as well as changes of the 3-dimensional structure represent functionally relevant chromatin stages [47]. Euchromatin is located towards the center of the nucleus and in this open form of chromatin, histone proteins are mostly acetylated and genomic DNA is unmethylated. In contrast, heterochromatin is found closer to the nuclear membrane and in this closed chromatin form, both nucleosomes and genomic DNA are methylated [48].

Epigenomics studies chromatin alterations that do not involve changes to the genome [49]. Epigenome changes, also referred to as epigenomic programing, are very prominent during embryogenesis, where totipotent stem cells beget various pluripotent cell lines of the embryo, which in turn act as precursors of terminally differentiated cells [50]. This differentiation process restricts the access to an increasing number of genomic regions and genes that they are controlling, so that terminally differentiated cells are able to focus on their specialized functions. Thus, chromatin accessibility plays an important role in regulating gene expression.

There is dynamic competition between nucleosomes and transcription factors for critical binding regions within genomic DNA, such as enhancers and promoters. Chromatin dynamics are influenced by a large set of chromatin modifying and remodeling enzymes, which interpret (“read”), add (“write”), or remove (“erase”) post-translational histone modifications or DNA methylation [51]. Interestingly, the activity of many of these chromatin modifiers critically depends on intracellular levels of key intermediary metabolites, such as NAD^+^, acetyl-CoA, and α-ketoglutarate [52]. In this way, environmental inputs, such as the availability of energy substrates, have direct effects on the epigenome and, by this, on gene expression. This implies that chromatin modifiers act as sensors of metabolic information, such as cells being in a fasting or feeding state.

The field of nutritional epigenomics describes numerous connections between diet-derived metabolites and the epigenome. For example, a number of secondary metabolites from fruits, vegetables, spices, teas, and medicinal herbs, such as resveratrol, genistein, curcumin, and polyphenols, affect the activity of chromatin modifiers and transcription factors [53]. Vitamin D and other micro- and macronutrients affect via their nuclear receptor sensors of chromatin accessibility, i.e., they belong to field of nutritional epigenomics [54]. Importantly, in contrast to epigenomic programing for cell fate decisions during cellular differentiation, which is largely irreversible, diet-induced epigenomic changes are dynamic, i.e., they are often transient and reversible.

## 6. Epigenomics of Vitamin D

The established model of vitamin D signaling [55] suggests that VDR, like RAR, PPAR, LXR, and other nuclear receptors [56], forms a heterodimer with the retinoid X receptor and preferentially binds to a DNA sequence that is formed of a direct repeat of two hexameric motifs spaced by three nucleotides, so-called DR3-type response elements [57]. The VDR cistrome [58], as obtained in a number of human cell lines, confirmed on a genome-wide level that DR3-type sequences are the most prominent VDR binding motifs. ChIP-seq data from B lymphocytes [59], monocytes [43,60], colorectal cancer cells [61], hepatic stellate cells [62], and macrophage-like cells [63] indicate that in the absence of ligand, VDR only binds to some 200–2000 sites per cell type. After stimulation with 1,25(OH)_2_D_3_, the number of VDR sites increases in average by a factor of 2.5 [63], which is the first direct epigenome-wide effect of vitamin D [54] (Figure 3B, I).

VDR is a “settler” type of transcription factor, i.e., it binds to a genomic region only when it finds its preferred sequence motifs within accessible chromatin. In contrast, “pioneer factors” [64] use DNA recognition sequences, which are short enough that they can be accessed even in the presence of nucleosomes. For example, VDR and the pioneer factors purine-rich box 1 (PU.1) and CCAAT/enhancer binding protein alpha (CEBPA) work closely together in hematopoietic differentiation into monocytes and granulocytes [37]. The pioneer factors PU.1 and CEBPA as well as GA binding protein transcription factor subunit alpha (GABPA) help VDR to find enhancer regions close to vitamin D target genes [65,66,67]. In THP-1 cells, 1,25(OH)_2_D_3_ modulates the binding of some 5–10% of PU.1, CEBPA, and GABPA binding sites, i.e., subsets of the cistromes of the respective pioneer factor are sensitive to the levels of the micronutrient. This represents the second level of epigenomic effects of vitamin D (Figure 3B, II).

At some 1300 genomic sites, the binding profile of the transcription factor CCCTC binding factor (CTCF) is also affected by 1,25(OH)_2_D_3_ [68]. CTCF is the key protein in organizing the human genome into loops, referred to as topologically associated domains (TADs) [69]. TADs are insulated from each other concerning gene regulatory effects, i.e., a transcription factor binding to an enhancer will modulate the expression of only those genes that have their transcription start sites (TSSs) within the same loop. In THP-1 cells, the vitamin D sensitivity of CTCF makes some 500 TADs dependent on VDR agonists, i.e., the modulation of the 3-dimensional organization of chromatin is the third level of the epigenome-wide effects of vitamin D (Figure 3B, III).

The high affinity binding of 1,25(OH)_2_D_3_ to the ligand-binding pocket within VDR’s ligand-binding domain causes a conformational change in the receptor protein [70]. This results in the loss of VDR’s contact with corepressor proteins, such as nuclear corepressor 1 (NCOR1) [71], which, in the absence of ligand, link the receptor to chromatin modifiers of the histone deacetylase family. Instead, in the presence of ligand, VDR interacts with co-activator proteins, such as those of the nuclear co-activator (NCOA) family [72], which either act themselves as histone acetyltransferases or form a complex with members of the respective chromatin modifier family. In addition, VDR communicates in a ligand-dependent fashion with chromatin modifiers of the lysine demethylase family, such as lysine demethylase 6B (KDM6B), or with chromatin remodelers like bromodomain containing 7 (BRD7). These proteins either form complexes with VDR [73,74] or their genes are primary targets of vitamin D [75]. The ligand-dependent interference of VDR with chromatin modifiers explains how vitamin D can affect histone markers for active TSSs, H3K4me3, and active chromatin, H3K27ac, as observed by ChIP-seq for these markers in 1,25(OH)_2_D_3_-stimulated THP-1 cells [66,76]. A change in histone modification has consequences on chromatin accessibility, as determined by FAIRE-seq [77], and represents the fourth level of epigenomic effects of vitamin D (Figure 3B, IV).

ChIP-seq and FAIRE-seq results obtained in 1,25(OH)_2_D_3_-triggered THP-1 cells are, to date, the most comprehensive set of data on epigenome-wide effects of vitamin D and were the most influential in formulating the chromatin model of vitamin D signaling [78]. This model is presently challenged in other cellular systems, such as in peripheral blood mononuclear cells (PBMCs), which were obtained from vitamin D_3_ bolus supplemented individuals [79].

## 7. Personalized Response to Vitamin D

The 1000 Genomes Project (www.internationalgenome.org) indicated that individuals differ from each other by in average by 4–5 million SNPs, some 0.5 million short insertions or deletions (indels), and up to a thousand larger copy number variations [80]. Although the majority of these variations are functionally neutral, there are many thousands of sites throughout the genome that increase the risk for common diseases or mediate the responsiveness to drugs. A well-known pharmacogenomics example of the latter is that of the anticoagulant drug warfarin, the individual’s responsiveness dependent on SNPs of the genes encoding for the enzymes vitamin K epoxide reductase complex subunit 1 (*VKORC1*) and cytochrome P450 family 2C9 (*CYP2C9*) [81]. Accordingly, there are high, mid, and low responders to warfarin and based on their genotype, individuals are prescribed different doses of the drug.

**Figure 4:**
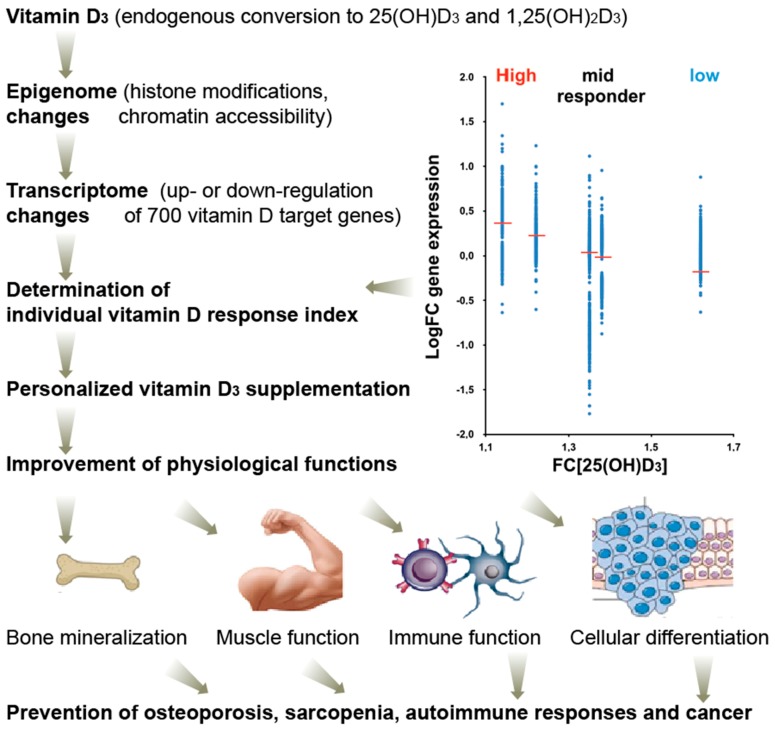
Clinical benefits of optimized vitamin D action. The vitamin D response index segregates individuals into high, mid, and low vitamin D responders and allows a more accurate monitoring of vitamin D effects in clinical setups, such as a prevention of osteoporosis, sarcopenia, autoimmune diseases, and possibly even cancer. The index is based on individual’s genetic and epigenetic status, but does not depend on their vitamin D status. It is determined primarily via changes of the transcriptome (i.e., mRNA transcription of vitamin D target genes) of vitamin D responding tissues, such as PBMCs. FC, fold change.

Recently, an analogous concept came up for vitamin D [82]. Based on the vitamin D_3_ supplementation studies VitDmet (NCT01479933, ClinicalTrials.gov) [83,84,85,86] and VitDbol (NCT02063334) [87,88], individuals show a personalized response to vitamin D_3_ and can be segregated into high, mid, and low responders. In contrast to in vitro cell culture experiments, supplementation studies have the advantage that they assess the activity of vitamin D under in vivo conditions. Thus, both types of intervention studies represent in vivo human nutrigenomic experiments. VitDmet was a long-term intervention study using daily vitamin D_3_ supplementation (0-80 µg) of pre-diabetic elderly individuals over 5 months of a Finnish winter, while VitDbol assessed the effects of a single vitamin D_3_ bolus (2000 µg) in young healthy adults already after one day. In both studies, PBMCs were isolated before and after supplementation and RNA and chromatin was prepared without any further in vitro culture of the cells. The index was initially determined on the basis of the response of 24 primary vitamin D target genes and 12 vitamin D sensitive biochemical parameters, such parathyroid hormone serum levels [86] as well as on the vitamin D-dependent change of the accessibility of a few chromatin regions [88], but most accurately, it was assessed by on the transcriptome-wide response of 702 vitamin D target genes (Figure 4, top right) [89]. In relation to fold changes in 25(OH)D_3_ serum levels, the individuals were ranked and classified into the three groups of vitamin D responsiveness. Both studies, VitDmet and VitDbol, indicated that some 25% of the participants are low responders, i.e., they display a low vitamin D response index [16]. Low vitamin D responders are most vulnerable against vitamin D deficiency. In order to keep vitamin D endocrinology at its optimal activity, low vitamin D responders need to take far higher daily vitamin D_3_ doses (maybe 50–100 µg) than high responders (10–20 µg).

The vitamin D response index was found to be independent of the 25(OH)D_3_ serum levels, i.e., of the vitamin D status of the study participants. In analogy to the warfarin responsiveness, it is assumed that the vitamin D response index is an intrinsic property of each individual and does not change during lifetime [82]. However, it is not yet known which genomic and epigenomic variations determine the index. The nutrigenomic assessment of in vivo vitamin D responsiveness, i.e., the determination of the vitamin D response index, implies that there should be a personalized vitamin D_3_ supplementation advice, rather than a general, population-based recommendation. Accordingly, the physiological responses to vitamin D on the level of bone and muscle health as well as on the prevention of autoimmune diseases and possibly different types of cancer (breast, prostate, and colon) are expected to be maximal (Figure 4, bottom).

## 8. Clinical Impact of Vitamin D Nutrigenomics

As outlined above, besides controlling proper bone mineralization, vitamin D’s main physiological role seems to be to the modulation of responses of the innate and adaptive immune responses. During the past years, a number of clinical studies demonstrated the immune system related benefit of vitamin D. For example, vitamin D deficiency in pregnancy may cause early asthma and wheezing, since vitamin D has effects on the developing lung and immune system during the fetal and early postnatal periods [90]. Therefore, vitamin D_3_ supplementation during pregnancy and infancy is important, in order to prevent aeroallergen sensitization, such as the proportion of children sensitized to mites, and the occurrence of respiratory illnesses, such as asthma [91]. Other randomized control trials indicated that vitamin D_3_ supplementation during pregnancy decreased the rate of respiratory tract infections or wheeze within the first 5 years of infant’s life [92]. Thus, a personalized vitamin D_3_ supplementation after vitamin D response index determination may reduce the predisposition toward developing allergic hypersensitivity reactions leading to asthma and/or respiratory tract infections more efficiently than relying exclusively on the vitamin D status. Moreover, vitamin D response index-based stratification of participants of future vitamin D intervention studies should lead to more convincing results concerning the benefits of vitamin D than reported recently for the risk of cancer and cardiovascular disease [93].

## 9. Conclusions

Molecular insights on nutrient-sensing systems allow a more integrative view of the reaction of the human body to dietary molecules. Vitamin D and its metabolites belong to a small set of dietary compounds that have direct effects on gene regulation. The analysis of vitamin D signaling via next generation sequencing technologies in in vitro cell culture models as well as in primary cells, such as PBMCs, has generated large amounts of data on the vitamin D-triggered epigenome and transcriptome in the respective cellular systems. Since the micronutrient connects cellular metabolism with immunity [94,95] (Figure 2C), nutrigenomics of vitamin D has a pleiotropic physiological and clinical impact. Personalized nutrition, such as tailored vitamin D supplementation, will contribute to the maintenance of wellbeing and the prevention of age- and lifestyle-related diseases, in particular those related to chronic inflammation.
